# Prähospitaler Kreislaufstillstand im Lockdown: Auswirkungen der übergreifenden Infektionspräventionsmaßnahmen während der ersten SARS-CoV-2-Welle

**DOI:** 10.1007/s10049-021-00932-7

**Published:** 2021-08-24

**Authors:** T. Grübl, B. Plöger, M. C. Sassen, A. Jerrentrup, B. Schieffer, S. Betz

**Affiliations:** 1 Zentrum für Notfallmedizin, Universitätsklinikum Gießen und Marburg, Standort Marburg, Baldingerstraße, 35043 Marburg, Deutschland; 2 Klinik für Anästhesie, Intensivmedizin, Notfallmedizin und Schmerztherapie, Bundeswehrzentralkrankenhaus, Rübenacher Straße 170, 56072 Koblenz, Deutschland; 3 Fachbereich Gefahrenabwehr, Landkreis Marburg-Biedenkopf, Im Lichtenholz 60, 35043 Marburg, Deutschland; 4 Klinik für Kardiologie, Angiologie und internistische Intensivmedizin, Universitätsklinikum Gießen und Marburg, Standort Marburg, Baldingerstraße, 35043 Marburg, Deutschland

**Keywords:** Kollaps, Reanimation, SARS-CoV‑2, COVID-19, Influenza, Collaps, Resuscitation, Severe acute respiratory syndrome coronavirus 2, Coronavirus disease 2019, Influenza

## Abstract

**Hintergrund:**

Das SARS-CoV‑2 (Severe acute respiratory syndrome coronavirus type 2) hat sich weltweit ausgebreitet. Folgen von Infektionspräventionsmaßnahmen im Rahmen solcher Ansteckungsereignisse können speziell für Patienten mit außerklinischem Kreislaufstillstand (OHCA) Nachteile ergeben.

**Methodik:**

Retrospektive Analyse von OHCA eines Landkreises in den Monaten Januar bis einschließlich Mai von 2018 bis 2020, wobei in 2020 die erste Welle der SARS-CoV-2-Pandemie und in 2018 eine Hochinzidenzphase des Influenzavirus vorlag.

**Ergebnisse:**

*N* = 497 OHCA wurden untersucht (2018 *n* = 173, 2019 *n* = 149, 2020 *n* = 175). Es zeigte sich eine gleichbleibende Reanimationsinzidenz (85–99 Reanimationen/100.000 Einwohner/Jahr) und eine lokal typische Klientel („mean“ 70 Jahre, 66 % männlich; Median PES 3). Es ergaben sich keine statistisch signifikanten Unterschiede bei der Ausgangslage der Patienten (Anzahl beobachteter OHCA, Häufigkeit an Laienreanimationen, vermutete Ursachen des OHCA, initialer EKG-Rhythmus) und dem Behandlungsverlauf (Häufigkeit an ROSC/Krankenhausaufnahme/Überleben bis Krankenhausentlassung, neurologisches Outcome). Keiner der OHCA-Patienten in 2020 bot ein positives SARS-CoV-2- und drei Patienten in 2018 ein positives Influenzatestergebnis.

**Diskussion:**

Die Lockdown-Maßnahmen während der ersten SARS-CoV-2-Welle scheinen das Outcome von OHCA-Patienten ohne COVID-19 insgesamt nicht beeinflusst zu haben.

## Abstract (en)

**Background:**

Severe acute respiratory syndrome coronavirus 2 (SARS-CoV-2) has spread worldwide. Consequences of infection prevention measures during such contagion events can cause disadvantages especially for patients in out-of-hospital cardiac arrest (OHCA).

**Methods:**

Retrospective analysis of OHCAs in one county from January–May in 2018, 2019 and 2020, with the first appearance of the SARS-CoV‑2 pandemic in 2020 and a high incidence of the influenza virus in 2018.

**Results:**

A total of 497 OHCAs were investigated (2018 *n* = 173; 2019 *n* = 149; 2020 *n* = 175). In this study, a constant resuscitation incidence (85–99 resuscitations/100,000 population/year) and locally typical patients (mean 70 years, 66% male; median PES 3) were found. There were no statistically significant differences in the initial situation of the patients (number of observed OHCAs, frequency of lay resuscitations, suspected causes of OHCAs, initial ECG rhythm) and the treatment course (frequency of return of spontaneous circulation [ROSC]/hospital admission/survival to hospital discharge, neurological outcome). None of the OHCA patients in 2020 tested positive for SARS-CoV‑2 and 3 patients in 2018 tested positive for the influenza virus.

**Discussion:**

The lockdown during the first wave of SARS-CoV‑2 pandemic does not seem to have affected the outcome of OHCA patients without coronavirus disease 2019 (COVID-19) in the end.

## Einleitung

Größere Infektionsgeschehnisse stellen die Medizin vor immense Herausforderungen. Vorerkrankte Patienten sind besonders gefährdet und das Gesundheitssystem wird stärker belastet. Ohne medizinisches Personal in solchen Situationen einem erhöhten Infektionsrisiko auszusetzen, müssen kritisch kranke Patienten schnell detektiert und behandelt werden. In der nachfolgenden Arbeit werden die Gegebenheiten außerklinischer Kreislaufstillstände während des ersten SARS-CoV-2-Lockdowns in einem Niedrigprävalenzbereich, während der letzten Influenzaepidemie und in einem Vergleichsjahr dargestellt.

Das in China im Dezember 2019 erstmalig beobachtete „severe acute respiratory syndrome coronavirus 2“ (SARS-CoV-2) hat sich weltweit ausgebreitet und verursacht die Erkrankung „coronavirus disease 2019“ (COVID-19; [3, 11]). Aus diversen Infektionsschutzstrategien resultieren unterschiedliche Auswirkungen auf das Gesundheitssystem. So vermeiden bspw. Patienten mit chronischen Leiden die Konsultation eines Arztes bzw. Krankenhauses, um sich dort nicht zu infizieren [6]. Dies könnte eine steigende Labilität des Grundleidens bedeuten und zu kritischen Situationen führen. Dabei wäre dann die Ablehnung oder Verzögerung von z. B. Wiederbelebungsmaßnahmen (kardiopulmonale Reanimation [CPR]) durch Laien, aus Angst vor Ansteckung, denkbar. Demgegenüber wäre das frühere Bemerken einer zunehmenden gesundheitlichen Verschlechterung oder eines Kollaps von Angehörigen möglich, da sich die Menschen im Rahmen von Kontaktvermeidungsstrategien oder eines angeordneten Lockdowns vorrangig zu Hause aufhalten.

Medizinisches Fachpersonal schützt sich bei potenziell kontagiösen Patienten grundsätzlich durch erweiterte Schutzausrüstung und führt bestimmte Maßnahmen wie die Reanimation oder Atemwegssicherung modifiziert durch [9, 10].

Solche veränderten Umstände können die Versorgung von Patienten mit außerklinischem Kreislaufstillstand („out-of-hospital cardiac arrest“ [OHCA]) relevant beeinflussen, deren sofortige Behandlung maßgeblich für das neurologische Outcome ist. Diese Arbeit vergleicht die Gegebenheiten bei der Versorgung von OHCA-Patienten während verschiedener Frühjahrssaisons; die SARS-CoV-2-Pandemie 2020 im ersten Lockdown, die jüngste Influenzaepidemie 2018 ohne solche Infektionspräventionsmaßnahmen in der Gesamtbevölkerung und vergleichend den Jahresbeginn 2019 ohne besondere Infektionsgeschehen.

## Methodik

Anhand einer retrospektiven und monozentrischen Studie wurden nach Zustimmung der Ethikkommission (Az.: ek_mr_19_11_2020_grübl, Ethikkommission Philipps-Universität Marburg, Fachbereich Medizin, 19.11.2020) alle OHCA-Patienten der Monate Januar bis einschließlich Mai in den Jahren 2018, 2019 und 2020 untersucht. Im Jahr 2018 lag im beschriebenen Zeitraum eine überdurchschnittlich hohe Influenzainfektionsrate vor und in 2020 trat in dieser Zeit die erste Welle von SARS-CoV‑2 auf.

Im beschriebenen Untersuchungsgebiet (1263 km^2^, 252.000 Einwohner, Bevölkerungsdichte 199/km^2^) existiert nur ein Cardiac Arrest Center und es wird studienunabhängig, abgeleitet von der Einsatzdokumentation des Notarztes sowie dem Krankenhausinformationssystem, ein zentrales Reanimationsregister geführt. Alle OHCA-Patienten wurden im Untersuchungszeitraum bei eventueller Krankenhausaufnahme in das Cardiac Arrest Center transportiert. Im Jahr 2020 erfolgte dort bei jedem Patienten im Rahmen der stationären Aufnahme ein nasopharyngealer Abstrich mit PCR-Untersuchung auf SARS-CoV‑2 und bei allen respiratorisch symptomatischen oder kritisch kranken Patienten eine thorakale Computertomographie. In 2018 erfolgte im Falle der stationären Aufnahme von Patienten mit Verdacht auf Influenza oder bei Risikopatienten für einen schweren Influenzaverlauf (chronische Lungenerkrankung, Dialysepflichtigkeit, Diabetes, Immunsuppression, neuromuskuläre Erkrankung oder BMI > 35 kg/m^2^) ein Influenzaschnelltest (ID Now^TM^ Influenza A&B 2).

Vor jedem Einsatz in 2020 legten Rettungsdienstmitarbeiter und Notarzt erweiterte persönliche Schutzausrüstung (FFP2-Maske, Augenschutz, Einmalkittel sowie doppelte Handschuhe) an. Weiter pausierte im kompletten Untersuchungszeitraum des Jahres 2020 das regionale First-Responder-System. In 2018 und 2019 existierten keine vom sonstigen Standard abweichenden Hygieneschutzmaßnahmen, medizinischen Vorgehensweisen, routinemäßigen Abstriche oder Alarmierungspläne.

Alle patientenbezogenen Behandlungsdaten wurden anonymisiert statistisch ausgewertet. Die Verwendung der anonymen Registerdaten erfolgte ohne direkte Einwilligung der Patienten.

Zur deskriptiven Statistik wurden je nach Skalenniveau und Verteilungsform der Mittelwert oder Median als zentrale Tendenz, Minima sowie Maxima als Extremwerte und Standardabweichungen sowie Interquartilsabstände als Dispersionsmaße berechnet. Anhand des Chi^2^-Vierfeldertests oder exakten Tests nach Fisher bei geringer Zellenbesetzung wurden die Häufigkeitsunterschiede auf Signifikanz geprüft. Die Signifikanzprüfung bei Unterschieden der zentralen Tendenz wurde für unabhängige Stichproben anhand des t‑Tests und für nichtparametrische mit dem Mann-Whitney-U-Test durchgeführt.

Bei einem grundsätzlich auf α = 0,05 festgesetzten Signifikanzniveau wurde zur Ergebnisinterpretation bei multiplem Testen eine Korrektur gemäß Bonferroni für abhängige Variablen vorgenommen [2].

## Ergebnisse

**Figure 1:**
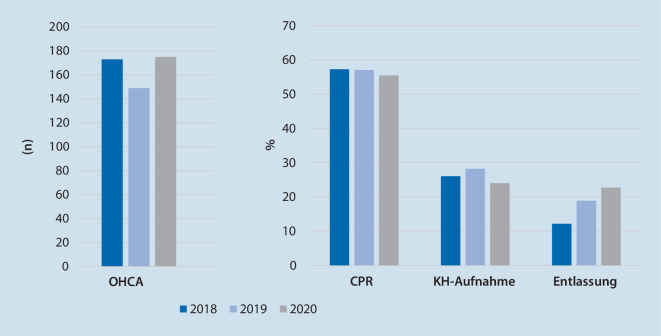

**Figure 2:**
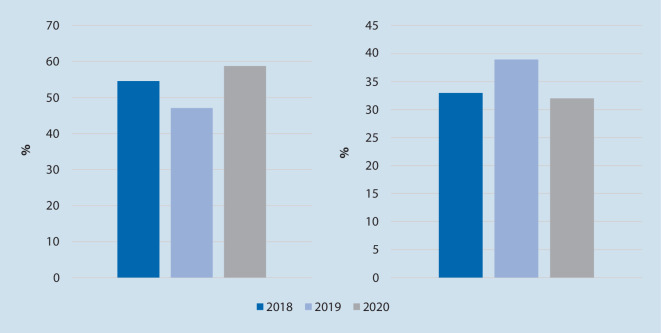

Vom 1. Januar bis 31. Mai der Jahre 2018, 2019 und 2020 wurden *n* = 497 Patienten, die einen OHCA erlitten, eingeschlossen. Die Rate an primären Todesfeststellungen bewegte sich jeweils auf einem vergleichbaren Niveau. Dies entspräche 85–99 Reanimationen/100.000 Einwohner pro Jahr. Die genauen Ergebnisse sind in Tab. 1 aufgeführt und anhand der Abb. 1, 2, 3 und 4 verdeutlicht.

**Table 1:** 

	*2018*		*2019*		*2020*		Vergleiche (*p*-Wert)		
	*n*	%	*n*	%	*n*	%	2018/19	2019/20	2018/20
**Basisdaten:**									
*OHCA*	173	–	149	–	175	–	–		
*CPR*	99	57,2	85	57,0	97	55,4	1,00	0,86	0,82
*Primäre Todesfeststellung*	74	42,8	64	43,0	78	44,6			
**Patientendaten:**									
*Alter, „mean“ (Jahre)*	70 (± 15)		69 (± 18)		70 (± 15)		0,89	0,80	0,93
*Geschlecht männlich*	104	60,0	103	69,0	120	69,0	0,12	1,00	0,12
*PES, Median*	3		3		3		0,71	0,93	0,54
**Einsatzdaten:**									
*Beobachteter Kollaps*	54	54,5	40	47,1	57	58,8	0,39	0,15	0,65
*Laienreanimation*	57	32,9	58	28,9	56	32,0	0,32	0,24	0,94
*EKG initial schockbar*	14	14,1	18	21,2	26	26,8	0,30	0,47	0,04
**Vermutete Ursache**									
Kardiales Ereignis	99	57,2	85	57,0	87	49,7	0,97	0,19	0,22
Hypoxie	30	17,4	19	12,8	30	17,2	0,32	0,35	1,00
Sonstige	44	25,4	45	30,2	58	33,1	0,41	0,65	0,14
*S4 bis Ankunft bei Pat., „mean“*	1,19 min (± 1,94)		1,14 min (± 1,98)		1,02 min (± 1,06)		0,93	0,90	0,83
**Verlaufsdaten:**									
*KH-Aufnahme*	45	26,0	42	28,2	42	24,0	0,70	0,50	0,87
Unter CPR	15	33,3	8	19,0	9	21,4	0,21	1,00	0,32
Mit ROSC	30	66,7	34	81,0	33	78,6			
*Entlassung*	12	12,1	16	18,8	22	22,7	0,29	0,65	0,08
**CPC**									
*„Mean“*	2		2		2		0,57	0,17	0,55
*1* *+* *2*	9	75,0	14	87,5	15	68,2	0,72	0,32	0,98

*CPC* Cerebral Performance Category,* CPR* kardiopulmonale Reanimation,* EKG* Elektrokardiogramm,* KH* Krankenhaus, *OHCA* „out-of-hospital cardiac arrest“,* Pat.* Patient,* PES* „Pre-emergency status“, *ROSC* „return of spontaneous circulation“,* S4* Uhrzeit bei Eintreffen erstes Rettungsmittel am Einsatzort

**Figure 3:**
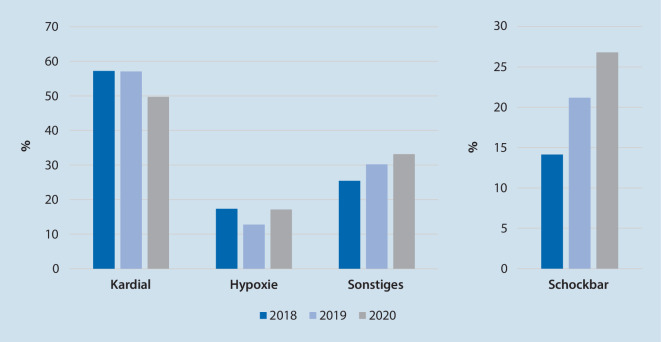

**Figure 4:**
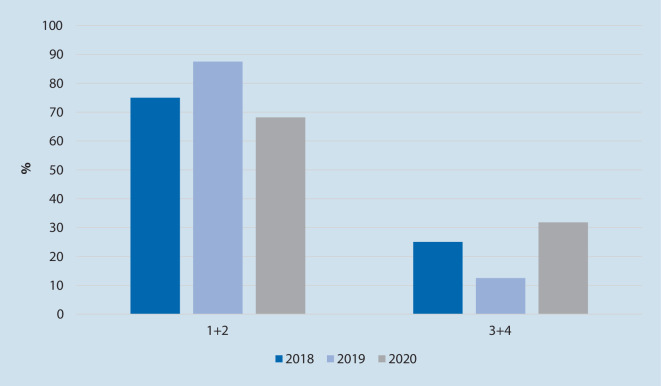

In den untersuchten Zeiträumen wurden keine statistisch signifikanten Unterschiede in der Anzahl an beobachteten OHCA, der Häufigkeit an Laienreanimationen, den vermuteten Ursachen des OHCA, den initialen EKG-Rhythmen, den Einsatzzeiten, der ROSC-Wahrscheinlichkeit, der Krankenhausaufnahme sowie der Überlebensrate bis zur Krankenhausentlassung und dem neurologischen Outcome gefunden.

In 2018 wurde bei 12 reanimierten Patienten ein Influenzaschnelltest durchgeführt, wobei drei ein positives Ergebnis boten, und bei zwei weiteren ergab sich radiologisch der Verdacht auf eine Viruspneumonie mit atypischen Infiltraten. Bei keinem der OHCA-Patienten aus 2020 ergab die standardgemäß nach Aufnahme im Schockraum durchgeführte Whole-body-Computertomographie atypische oder COVID-typische pulmonale Infiltrate. Auch die Polymerase-Kettenreaktion (PCR-Untersuchung) der nasopharyngealen Abstriche ergab keine SARS-CoV-2-Nachweise. Von Januar bis einschließlich Mai 2020 lag im beschriebenen Untersuchungsgebiet die durchschnittliche 7‑Tage-Inzidenz von SARS-CoV‑2 bei 10,4/100.000 Einwohner, insgesamt wurden 10 Patienten auf einer Intensivstation behandelt und es wurden insgesamt 3 Todesfälle in Zusammenhang mit SARS-CoV‑2 registriert.

## Diskussion

Insgesamt wurde in den untersuchten Zeitfenstern der drei Jahre eine lokal übliche Anzahl an OHCA bei einer ebenso üblichen Patientenklientel in Bezug auf Alter, Geschlecht und Vorerkrankungsstatus gefunden. Es zeigten sich keine statistisch signifikanten Unterschiede bei der Rate an beobachteten OHCA, Laienreanimationen, Krankenhausaufnahmen sowie Entlassungen. Auch der neurologische Status bei Entlassung unterschied sich nicht statistisch signifikant. Die Anzahl an schockbaren EKG-Rhythmen bei der initialen Analyse stieg von 2018 auf 2020 um 13 % (*p* = 0,04; Tab. 1). Bei multipler Testung im gleichen Datensatz ergab sich nach Korrektur jedoch keine statistische Signifikanz (α* < 0,0125).

Bei den hypothetischen Überlegungen über eventuelle Nachteile für Patienten mit außerklinischem Kreislaufstillstand aufgrund der aktuellen politisch-gesellschaftlich und medizinisch eingeleiteten Maßnahmen zur Pandemieeindämmung und Infektionsprävention deuten die hiesigen Ergebnisse also auf eher gleichbleibende Behandlungsverläufe hin. Da bei keinem der Patienten abschließend eine SARS-CoV-2-Infektion nachgewiesen wurde, betrifft dies bisher nur Patienten, die aus anderweitigen Ursachen einen außerklinischen Kreislaufstillstand erlitten.

Zwar ist das Patientenkollektiv klein und es existiert keine direkte Vergleichsgruppe in dieser Arbeit, jedoch können durch solche Erkenntnisse die bestehenden Modifikationen von Vorgehensweisen im Gesundheitssystem hierzulande weiter unterstützt werden.

In Niedrigprävalenzgebieten scheint sich dieses Bild, den Kollegen Elmer et al. aus diesem Jahr zu Folge, auch an anderer Stelle zu bestätigen [4].

Jedoch zeigen sich in der vorliegenden Untersuchung einige Trends. In 2018 und 2020 ereigneten sich etwas mehr außerklinische Kreislaufstillstände. Bereits in der Einleitung wurde auf eine eventuelle Zustandsverschlechterung vorerkrankter Patienten hingedeutet, auch unabhängig von einer COVID-19-Erkrankung. Weiter lag eine höhere Rate an beobachteten OHCA vor, was aus der Hypothese der gesteigerten Präsenz der Menschen in ihren Wohnungen abgeleitet werden könnte. Dies wäre ebenso eine Erklärung für die häufiger diagnostizierten schockbaren EKG-Rhythmen, da der Notarzt im Sinne eines beobachteten Kreislaufstillstand wahrscheinlich zügiger alarmiert wurde und so auch die Prognose günstig beeinflusst wird [5, 7].

Gemäß der oben aufgestellten Hypothese wurden in den Infektionslagen von 2018 und 2020 trotzdem etwas weniger Laienreanimationen durchgeführt. Da keine systematische Nachbefragung der Laienhelfer erfolgte, lassen sich Gründe, wie die Angst vor Ansteckung, nur vermuten.

Bei gleichbleibender Rate an durchgeführten Reanimationen durch den Rettungsdienst wurden trotzdem weniger Patienten ins Krankenhaus transportiert. Eventuelle Gründe hierfür könnten Hemmung gegen den Transport unter Isolationsbedingungen bei nicht auszuschließender SARS-CoV-2- bzw. Influenzainfektion oder eine schlechtere Prognoseeinschätzung aufgrund von prolongierten oder modifizierten Maßnahmen während der präklinischen Reanimation sein. Trotzdem konnte eine steigende Zahl an Krankenhausentlassungen nach OHCA über die Studienzeiträume verzeichnet werden. Das häufigere Auftreten von schockbaren EKG-Rhythmen ist eine denkbare Ursache. Ebenso könnten aber auch der fortwährende Optimierungsprozess bestehender Leitlinien mit differenzierten Entscheidungspfaden und zunehmendes Training von notfallmedizinischem Personal, unabhängig vom Infektionsgeschehen, ihre angestrebte Wirkung zeigen.

Auch wenn ebenso keine statistische Signifikanz vorlag, zeigten sich mehr Patienten mit einem schlechteren neurologischen Outcome (CPC 3 + 4). Ob bei schlechter Ausgangsprognose eine frühere Therapieeinleitung bei häufigeren schockbaren EKG-Rhythmen oder der Ausfall des First-Responder-Systems hier ursächlich ist, kann an dieser Stelle nicht abschließend geklärt werden.

Die Angaben über die Dauer vom Eintreffen an der Einsatzstelle bis zur Ankunft beim Patienten zeigten keine größeren Schwankungen, trotz des vorigen Anlegens von erweiterter persönlicher Schutzausrüstung im Jahr 2020. Da speziell dieses Zeitintervall vom präklinischen Personal selten genau aufgezeichnet, sondern mutmaßlich eher geschätzt wird, könnte sich das hiesige Ergebnis als nicht reliabel erweisen oder auf die gute Vorbereitung und Organisation des Anlegens von Schutzausrüstung im Einsatz hindeuten.

Andere Studien aus schwerer betroffenen Pandemiegebieten der Welt zeigen deutlich gestiegene OHCA-Inzidenzen und reduzierte Überlebenswahrscheinlichkeiten. Diese Ergebnisse werden dort jedoch als klar SARS-CoV-2- bzw. COVID-19-korreliert beschrieben [1, 8].

Der Vergleich der letzten Influenzaepidemie und der jetzigen SARS-CoV-2-Pandemie ist dabei brisant sowie interessant, da diese beiden Geschehnisse und die dabei getroffenen Infektionspräventionsmaßnahmen mitunter vergleichend gegenübergestellt und bewertet werden.

Die hier gewonnen Ergebnisse sollten also abschließend als Hinweise aus einem Niedrigprävalenzbereich in Bezug auf allgemeine OHCA-Patienten ohne SARS-CoV-2-Infektion interpretiert werden. Um die verschiedenen Einflüsse genauer und über einen längeren Zeitraum bewerten zu können, sind weitere Untersuchungen bei insgesamt gestiegener COVID-19-Inzidenz notwendig und werden von den Autoren in dem hier beschriebenen Bereich aktuell konzeptioniert.

## Fazit für die Praxis

- Die ergriffenen Infektionsschutzmaßnahmen im Rahmen der SARS-CoV-2-Pandemie scheinen in Niedrigprävalenzgebieten zum aktuellen Zeitpunkt keine größeren Auswirkungen auf den Behandlungsverlauf von Patienten mit außerklinischem Kreislaufstillstand ohne SARS-CoV-2-Infektion zu haben.
- In schwerer betroffenen Gebieten der SARS-CoV-2-Pandemie zeigte sich jedoch ein deutlicher Anstieg der OHCA-Inzidenz sowie eine Outcomeverschlechterung.
- Medizinisches Fachpersonal sollte nach wie vor den Eigenschutz in größeren Infektionslagen priorisieren und darunter eine leitlinienkonforme Behandlung anstreben.

## References

[1] Baldi E, Sechi GM, Mare C, Canevari F, Brancaglione A, Primi R, Klersy C, Palo A, Contri E, Ronchi V, Beretta G, Reali F, Parogni P, Facchin F, Rizzi U, Bussi D, Ruggeri S, Oltrona Visconti L, Savastano S (2020). COVID-19 kills at home. The close relationship between the epidemic and the increase of out-of-hospital cardiac arrests. Eur Heart J.

[2] Bortz J (2008). Kurzgefasste Statistik für die klinische Forschung. Leitfaden für die verteilungsfreie Analyse kleiner Stichproben.

[3] Dong E, Du H, Gardner L (2020). An interactive web-based dashboard to track COVID-19 in real time. Lancet Infect Dis.

[4] Elmer J, Okubo M, Guyette FX, Martin-Gill C (2020). Indirect effects of COVID-19 on OHCA in a low prevalence region. Resuscitation.

[5] Hansen CM, Kragholm K, Granger CB, Pearson DA, Tyson C, Monk L, Corbett C, Nelson RD, Dupre ME, Fosbøl EL, Strauss B, Fordyce CB, McNally B, Jollis JG (2015). The role of bystanders, first responders, and emergency medical service providers in timely defibrillation and related outcomes after out-of-hospital cardiac arrest. Results from a statewide registry. Resuscitation.

[6] Hartnett KP, Kite-Powell A, DeVies J, Coletta MA, Boehmer TK, Adjemian J, Gundlapalli AV (2020). Impact of the COVID-19 pandemic on emergency department visits – United States, January 1, 2019–May 30, 2020. MMWR Morb Mortal Wkly Rep.

[7] Ko SY, Shin SD, Ro YS, Song KJ, Hong KJ, Park JH, Lee SC (2018). Effect of detection time interval for out-of-hospital cardiac arrest on outcomes in dispatcher-assisted cardiopulmonary resuscitation. A nationwide observational study. Resuscitation.

[8] Marijon E, Karam N, Jost D, Perrot D, Frattini B, Derkenne C, Sharifzadehgan A, Waldmann V, Beganton F, Narayanan K, Lafont A, Bougouin W, Jouven X (2020). Out-of-hospital cardiac arrest during the COVID-19 pandemic in Paris, France. A population-based, observational study. Lancet Public Health.

[9] Nolan JP, Monsieurs KG, Bossaert L, Böttiger BW, Greif R, Lott C, Madar J, Olasveengen TM, Roehr CC, Semeraro F, Soar J, van de Voorde P, Zideman DA, Perkins GD (2020). European Resuscitation Council COVID-19 guidelines executive summary. Resuscitation.

[10] World Health Organization (2020) Prevention, identification and management of health worker infection in the context of COVID-19. Interim guidance. COVID-19: Essential health services, 30 October 2020. https://www.who.int/publications/i/item/10665-336265. Zugegriffen: 10. Nov. 2020

[11] Wu Y-C, Chen C-S, Chan Y-J (2020). The outbreak of COVID-19. An overview. J Chin Med Assoc.

